# Preparation and Characterization of α-Zinc Molybdate Catalyst: Efficient Sorbent for Methylene Blue and Reduction of 3-Nitrophenol

**DOI:** 10.3390/molecules23061462

**Published:** 2018-06-15

**Authors:** Hicham Oudghiri-Hassani, Souad Rakass, Mostafa Abboudi, Ahmed Mohmoud, Fahd Al Wadaani

**Affiliations:** 1 Chemistry Department, College of Science, Taibah University, Al-Madinah 30002, Saudi Arabia; rakass_souad@yahoo.fr (S.R.); abboudi14@hotmail.com (M.A.); caadil77@yahoo.co.uk (A.M.); fwadaani@taibahu.edu.sa (F.A.W.); 2 Département Sciences de la Nature, Cégep de Drummondville, 960 rue Saint-Georges, Drummondville, QC J2C 6A2, Canada

**Keywords:** zinc molybdate, nitrophenol reduction, sorbent, methylene blue, nanoparticles

## Abstract

Zinc molybdate (ZnMoO_4_) was prepared by thermal decomposition of an oxalate complex under a controlled temperature of 500 °C. Analyses of the oxalate complex were carried out using Fourier transform infrared spectroscopy (FTIR) and thermogravimetric analysis (TGA). On the other hand, analyses of the synthesized zinc molybdate were carried out by X-ray diffraction (XRD), transmission electron microscopy (TEM), and Brunauer-Emmett-Teller technique (BET). The efficiency of the synthesized catalyst was tested with the reduction reaction of 3-nitrophenol (3-NP), and was also applied as a sorbent for methylene blue dye (MB) in aqueous solutions. The catalytic test of zinc molybdate shows a very high activity. The concentration reduction progress and adsorption of the dye were followed by an ultraviolet-visible (UV-vis) spectrophotometer.

## 1. Introduction

Zinc molybdate is among the most studied ternary oxides in the family AMoO_4_ (A is a transition element or a divalent metal from the alkaline earth column). It exists in two crystallographic varieties, the α-ZnMoO_4_, which has a triclinic cell where the zinc is in an octahedral site and the molybdenum is in a tetrahedral site, and the β-ZnMoO_4_, that is monoclinic with both of zinc and molybdenum in octahedral geometry. ZnMoO_4_ is not soluble in water, which reduces its toxicity. It is used as a white pigment [1]. This material was used in a variety of applications, such as photocatalysis [2,3,4,5], as phosphor for light emitting diode [6,7,8,9,10,11,12,13], electrochemical anode material for lithium batteries [14], in scintillating bolometers for double beta decay of ^100^Mo [15,16], humidity sensors [17], antibacterial [18], biological imaging of deep tumors [19], supercapacitor [20], and as catalyst in the oxidation of propane and propene [21,22]. Traditionally, the zinc molybdate varieties are prepared by a conventional solid-state reaction [8,12], hydrothermal or solvothermal [2,5,7,10,11,19,20], mechanochemical [23], combustion synthesis [24], co-precipitation [3,19,25], sol-gel [26], or sonochemical method [6]. To our knowledge, there is no investigation reported on the use of zinc molybdate as catalyst in the reduction of nitrophenol to aminophenol, and neither in dye removal from polluted industrial waters. The reduction of the nitro group to the amine group is an important step in the synthesis of chemicals and drugs. For example, the reduction of the para-nitrophenol produces the para-aminophenol, which is used in the synthesis of the paracetamol (acetaminophen) drug [27]. On the other hand, water remediation is a concern of researchers across the world. Many efforts are made in order to replace the use of the activated carbon by materials having the same or better efficiency in dye removal by adsorption from polluted industrial waters and using low-cost materials. For this purpose, materials from natural products were largely investigated, such as agricultural wastes [28,29,30] or clay minerals [31,32,33]. Research on de-colorization was also focused, for example, on the use of metals oxides [34,35] or composite materials [36,37,38].

Therefore, in this contribution, we report the synthesis of the α-ZnMoO_4_ nanomaterial using a different method that uses precursors, such as oxalate complexes of zinc and molybdenum. The prepared α-ZnMoO_4_ nanoparticles were investigated for two applications: for their catalytic efficiency in the reduction of the 3-nitrophenol (3-NP) to the 3-aminophenol (3-AP), and for their efficiency in dye removal from polluted waters. The methylene blue was taken as a standard dye. 

## 2. Experimental

### 2.1. Preparation of Zinc Molybdate

Zinc molybdate was prepared by first grinding together oxalic acid H_2_C_2_O_4_·2H_2_O, ammonium molybdate (NH_4_)_6_Mo_7_O_24_·4H_2_O, and zinc nitrate Zn(NO_3_)_2_·6H_2_O at a ratio of 10/0.143/1 [39]. This mixture produced an oxalate precursor at a temperature of 160 °C. All the chemical reagents were of analytical grade, purchased from Sigma-Aldrich, and were used in their received state.

Reduction of nitrate and molybdenum anions was carried out with an excess of oxalic acid (Equation (1)), in order to form a coordinated complex of zinc and molybdenum. The complex formation and redox reactions were established by heating, in their solid state, on a hot plate. When nitrate anion (NO_3_^−^) and molybdenum VI were reduced at a temperature of 160 °C (Equations (2) and (3)), a brown-orange evolving gas and pale-blue powder were observed. This was due to the formation of NO_2_ gas (Equation (4)) and zinc molybdenum complex, respectively. Finally, the zinc molybdenum complex was thermally decomposed in order to form the zinc molybdate by heating in static air under a temperature of 500 °C inside a tubular furnace opened at both ends [40,41,42,43].

C_2_O_4_^2−^ →2CO_2_ + 2 e^−^ (1)

Mo^VI^ (white) + 2 e^−^ →Mo^IV^ (blue) (2)

NO_3_^−^ + 4 H^+^ +3 e^−^ →NO + 2H_2_O (3)

NO + ½ O_2_ →NO_2_ (brown-orange) (4)

### 2.2. Analysis and Characterization of Zinc Molybdate

Thermal analysis of the prepared zinc molybdenum complex was carried out by thermogravimetric analysis (TGA, SQT 600, Ta Instruments, New Castle, DE, USA). Characterization was established using FTIR (Shimadzu 8400S, Tokyo, Japan) in the range of 400–4000 cm^−1^, preparing the sample as a KBr pellet. Adsorption–desorption isotherms and particle sizes were measured using Micromeritics ASAP 2020 surface area and porosity analyzer (Micromeritics, Norcross, GA, USA), with the equation D_BET_ = 6000/d.S, where d is the density, and S is the specific surface area. The crystalline nanoparticles, in the range of 10° to 80° in 2θ of the sample, were identified by X-ray diffractometer 6000 (Shimadzu, Tokyo, Japan) installed with λ_Cu-Kα_ = 1.5406 Ǻ and Ni filter. The spherical particle size was worked out using the Scherer equation D_XRD_ = 0.9 λ/(B cosθ), where B is the full width at half maximum (FWHM) expressed in radians, λ is the Cu–Kα wavelength, and θ is the Bragg angle. During the adsorption of methylene blue dye and the reduction reaction of 3-nitrophenol (3-NP), the solution concentration was measured using Varian Cary 100 spectrophotometer (Varian Inc, Palo Alto, CA, USA), and the shape and particle size were calculated using JEM-1400 electron microscope (JOEL, Peabody, MA, USA).

### 2.3. 3-Nitrophenol Reduction Test

The catalytic activity of zinc molybdate was tested with the reduction of 3-NP. The test solution included an aqueous solution mixture consisting of 40 mL of 4 × 10^−4^ M 3-NP with 40 mL of 8 × 10^−4^ M sodium tetrahydroborate (NaBH_4_), stirring steadily at room temperature. The presence of nitrophenolate ion was observed by the visual appearance of a dark yellow color and an absorption peak at 393 nm for the 3-NP. The effect of the zinc molybdate catalyst was investigated with the UV-vis spectrophotometer when 0.1 g of the catalyst was added to the solution mixture, which resulted in the disappearance of the dark yellow color.

### 2.4. Adsorption Test

Solutions of different concentrations of methylene blue dye for the adsorption test were prepared from a stock solution of 1000 mg·L^−1^. Adsorption of methylene blue by zinc molybdate nanoparticles was conducted by adding 0.1 g of the ZnMoO_4_ into 100 mL of methylene blue solution. The pH of the solution was adjusted before the addition, to pH = 3. Solutions were filtered by Thermo Scientific Syringe Filters in 4 mm, 17 mm, and 30 mm diameters with pore size of 0.2 μm. The residual methylene blue concentration was analyzed with UV-vis spectrophotometer at λ_max_ = 665 nm. The removed percentage (%) of methylene blue and the amount adsorbed at equilibrium (q_e_) were calculated with the following equations accordingly:

(5) $$\;\mathrm{Removal}\;\text{\%}\;=\;\frac{C_{0}-C_{e}}{C_{0}}\times 100$$

(6) $$\;q_{e}=\;\frac{\left(C_{0}-C_{e}\right)}{W}\;\times \;V$$

where q_e_ (mg·g^−1^) is the amount of the MB dye adsorbed at equilibrium by the synthesized zinc molybdate nanoparticle, C_0_ is the initial dye concentration (mg·L^−1^), C_e_ is the dye concentration at equilibrium (mg·L^−1^); W is the mass of the adsorbent (g), and V is the volume of the solution (L).

### 2.5. Desorption Test

The desorption of MB was studied on a sample obtained from the adsorption in the case of the aqueous solution of 130 ppm of MB at pH = 5, to get an idea about the recycling possibility and the reuse of the catalyst. The experiment was done by putting 0.1g of the filtered catalyst in 100 mL of a solution of ethanol/water (50/50) maintained under stirring at room temperature. The variation of the concentration of the desorbed MB in the solution was followed by UV visible spectrophotometer. The desorption rate was calculated by the following equation:

(7) $$\mathrm{Desorption}\%=\;\frac{C_{des}}{C_{ads}}\times 100$$

where C*_des_* is the amount of the MB dye desorbed by the solution of ethanol/water (50/50), and C*_ads_* is the amount of the MB dye adsorbed in the case of the aqueous solution of 130 ppm of MB at pH = 5.

## 3. Results and Discussion

### 3.1. Complex Identfication and Characterization

The product of the reaction in the solid state mixture of oxalic acid, zinc nitrate, and ammonium molybdate, which was ground and calcinated at 160 °C, showed the functional groups identified by FTIR spectroscopy.

**Figure 1:**
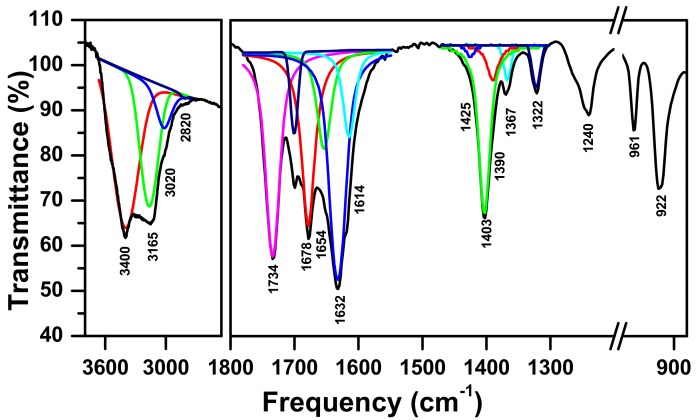
The FTIR spectrum for the mixture of oxalic acid H_2_C_2_O_4_·2H_2_O at 160 °C, ammonium molybdate (NH_4_)_6_Mo_7_O_24_·4H_2_O, and nickel nitrate Zn(NO_3_)_2_·6H_2_O.

A variety of wide bands can be seen from the IR spectrum in Figure 1. The constituents of these bands show that assignments at 1734 cm^−1^ and 1678 cm^−1^ are given to the C=O vibration of the oxalate group [44]. This is in agreement with the C–O stretch [44] at 1403 cm^−1^. On the other hand, the bands at 1322 and 1367 cm^−1^ are distinctive to δ(OCO) and υ(C–O), respectively [45].

Ammonia and ammonium ion are both present via the deformation modes of the asymmetric and symmetric bands at 1614 cm^−1^ δ_s_(NH_3_), 1240 cm^−1^ δ_as_(NH_3_), and at 1654 cm^−1^ δ_s_(NH_4_^+^), 1425 cm^−1^ δ_as_(NH_4_^+^), respectively. It can also be seen that the bands at 3020 cm^−1^ and 2820 cm^−1^, and at 3165 cm^−1^ in the NH stretching region, are identified as the ammonium ions and coordinated ammonia, respectively. The findings of the study agree with Ramis et al. [46] and Wen et al. [47]. There are two metals with a bridging group of O–H, which can be seen at 3400 cm^−1^ [48,49]. The absorbance spectrum for the δ(OH) is shown at 1390 cm^−1^ [49], whereas the band at 1632 cm^−1^ is assigned to δ(H_2_O) [50]. Furthermore, the bands at 961 cm^−1^ and 922 cm^−1^ distinguish the presence of the Mo=O stretch [44]. Therefore, it can be confirmed that the synthesized complex contains functional groups such as water, NH_3_, oxalate, NH_4_ ion, hydroxyl (–OH), and oxo (Mo=O).

**Figure 2:**
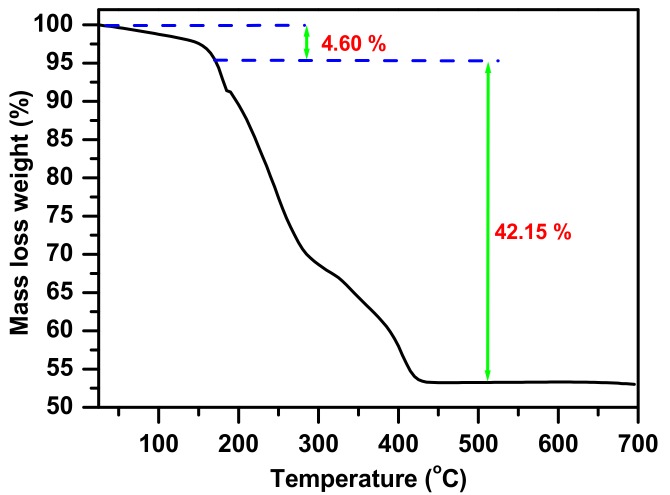
Thermogravimetric curve for the complex consisting of an oxalic acid H_2_C_2_O_4_·2H_2_O, ammonium molybdate (NH_4_)_6_Mo_7_O_24_·4H_2_O, and zinc nitrate Zn(NO_3_)_2_·6H_2_O at a temperature of 160 °C.

Studies of the thermogravimetric analysis (Figure 2) were carried out on the synthesized complex under static air. The obtained curve shows the distinction of three parts. The decline of the curve up to 170 °C is due to the weight loss of 4.6%, which can be explained as the loss of water molecules in the synthesized complex, and this agrees with the confirmation of the reported infrared spectroscopy. The remaining parts of the curve show a fast loss from 170 °C to 440 °C, this is due to the decomposition of the synthesized complex with a weight loss of 42.15%. After careful studies, collection of the results from TGA and FTIR, with the oxidation degree of molybdenum and zinc, the suggested formula can be concluded as (NH_3_)(NH_4_)ZnMoO(C_2_O_4_)_2_(OH)·H_2_O. The theoretical figure of 46.75% is in agreement with the suggested formula with a total weight loss of 46.75%. The zinc molybdate complex was obtained via calcination at a carefully chosen constant temperature of 500 °C.

### 3.2. Zinc Molybdate Characterization

#### 3.2.1. X-ray Diffraction

**Figure 3:**
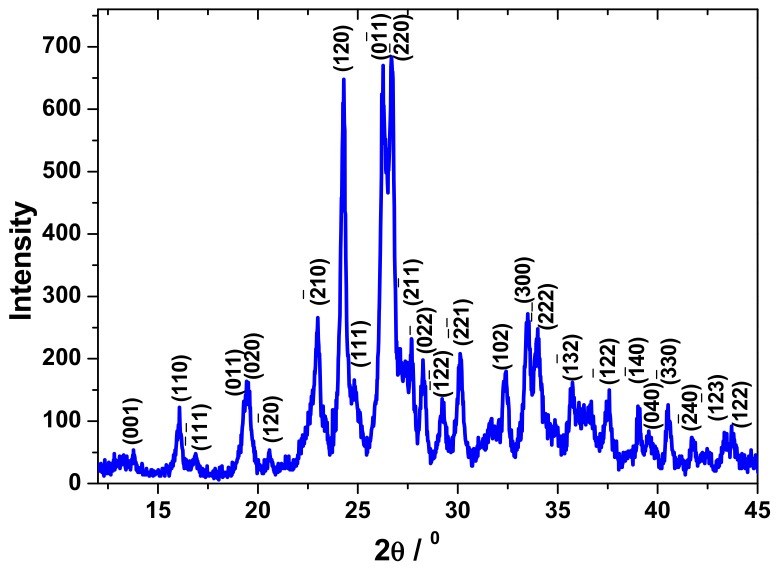
X-ray diffraction pattern of the synthesized zinc molybdate after calcination the oxalate complex at a temperature of 500 °C.

The X-ray diffraction (XRD) technique was used to analyze the end-produced complex after calcination at 500 °C, and the presented pattern shown in Figure 3 was recorded. The XRD pattern agrees with the JCPDS #35-0765 index file. This matches with the triclinic phase α-ZnMoO_4_ that crystallizes in the space group P $\bar{1}$(2) with parameters a = 8.3678(8), b = 9.6916(8), and c = 6.9643(6), and α =106.872(8), β = 101.726(8), and γ = 96.734(8).

The highest d spacing which was chosen to calculate the crystallite size, and D_XRD_ was the intense peak located at 2θ = 24.18° (120), and this gives a value for D_XRD_ to be of 24 nm.

#### 3.2.2. Specific Surface Area Determination

Brunauer-Emmett-Teller (BET) method [51] was used to find out the specific surface area of the synthesized zinc molybdate, ZnMoO_4_. With the density value of the zinc molybdate being d = 4.3 g/cm^3^, it was found out that the S_BET_ = 18.9 m^2^/g, and the particle size D_BET_ was calculated to be approximately of 74 nm. Barrett, Joyner, and Halenda (BJH) method calculations allows us to find out that the pore volume was 0.1428 cm^3^/g with a pore size of 297 Å. This indicates that the material has mesoporous characteristics [52].

#### 3.2.3. Transmission Electron Microscopy

**Figure 4:**
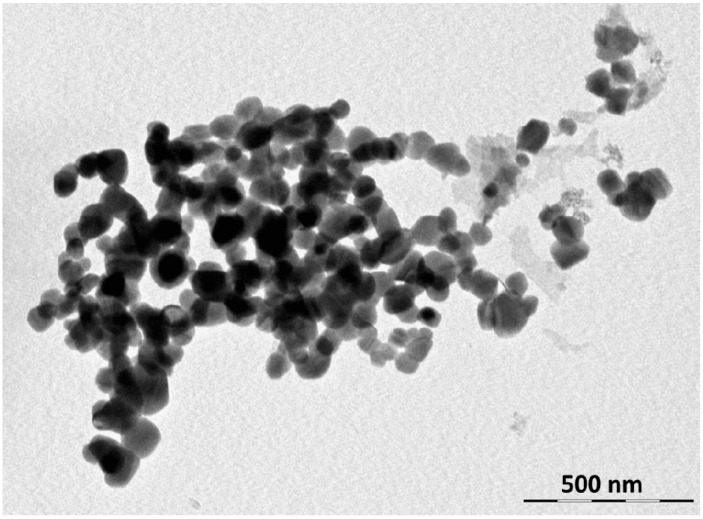
Micrograph of the transmission electron microscopy for the synthesized zinc molybdate, ZnMoO_4_, gained after calcination of the oxalate complex at a temperature of 500 °C.

Figure 4 shows a synthesized zinc molybdate micrograph. The particles are between 70 to 100 nm in size, and are in a spherical shape. The particle size observed from BET, 74 nm, is in accordance with that found in transmission electron microscopy (TEM), 70–100 nm. However, the X-ray diffraction analysis does not confirm the same value. The value, 24 nm, observed in the XRD analysis where the crystallite size is reached, is different from particle size. When the particles agglomerate, they form particles of greater size, lowering the adsorption rate of the liquid nitrogen molecules. The resulting specific surface area value will decrease, giving higher particle sizes than expected. On the other hand, the crystallite size is measured in XRD, even if the powder is agglomerated into larger particles, because the crystallites are separately crystallized.

### 3.3. 3-Nitrophenol Reduction Test

**Figure 5:**
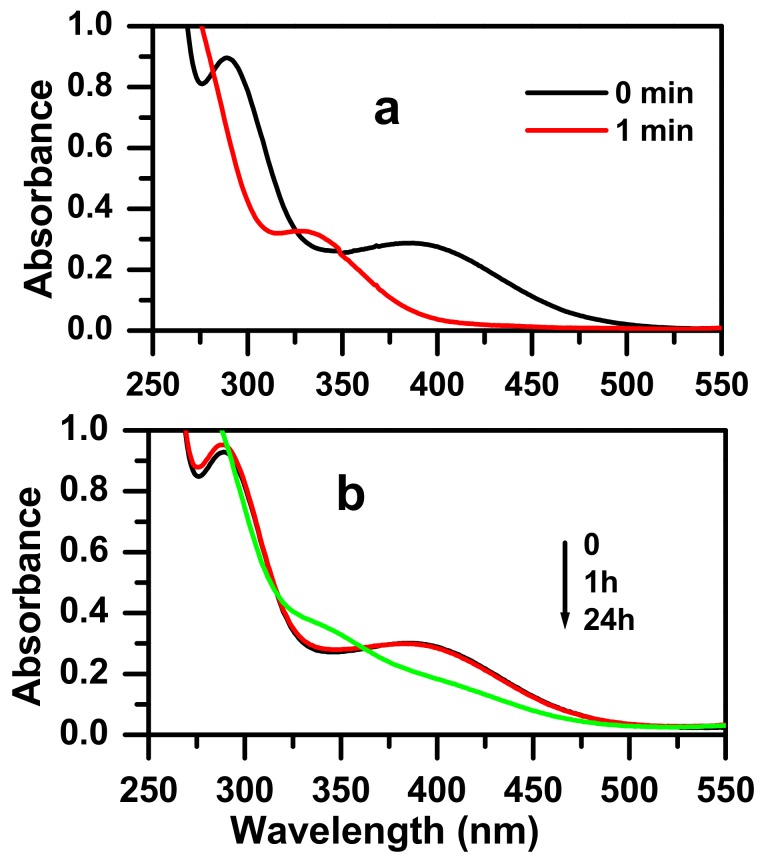
The UV-visible spectra for the reduction reaction solution of 3-NP in the presence of NaBH_4_ at room temperature (**a**) after the addition of zinc molybdate, ZnMoO_4_, prepared by calcination of the oxalate complex at a temperature of 500 °C, and (**b**) without adding the catalyst.

The synthesized zinc molybdate was tested to investigate the catalytic efficiency for the reduction reaction of 3-nitrophenol, 3-NP, against NaBH_4_ (Figure 5). After the addition of NaBH_4,_ the 3-NP converted to 3-NP ion nitrophenolate. The dark yellow color of the 3-NP solution disappears in few minutes after the addition of the zinc molybdate nanocatalyst, whilst for the addition of the as-prepared catalyst alone, the color of the solution did not change, even for a period of over 24 h. The UV-vis absorption peak comparison shows that the higher peak of absorption situated at 393 nm disappears in favor to a new peak appearing at 328 nm. The observed time taken for the complete reaction with the appearance of the corresponding 3-aminophenol (3-AP), at room temperature, was around 1 min. The results confirmed the high catalytic efficiency of the synthesized zinc molybdate in the reduction of 3-nitrophenol compared to previously reported research work in the literature, as shown in Table 1.

**Figure 6:**
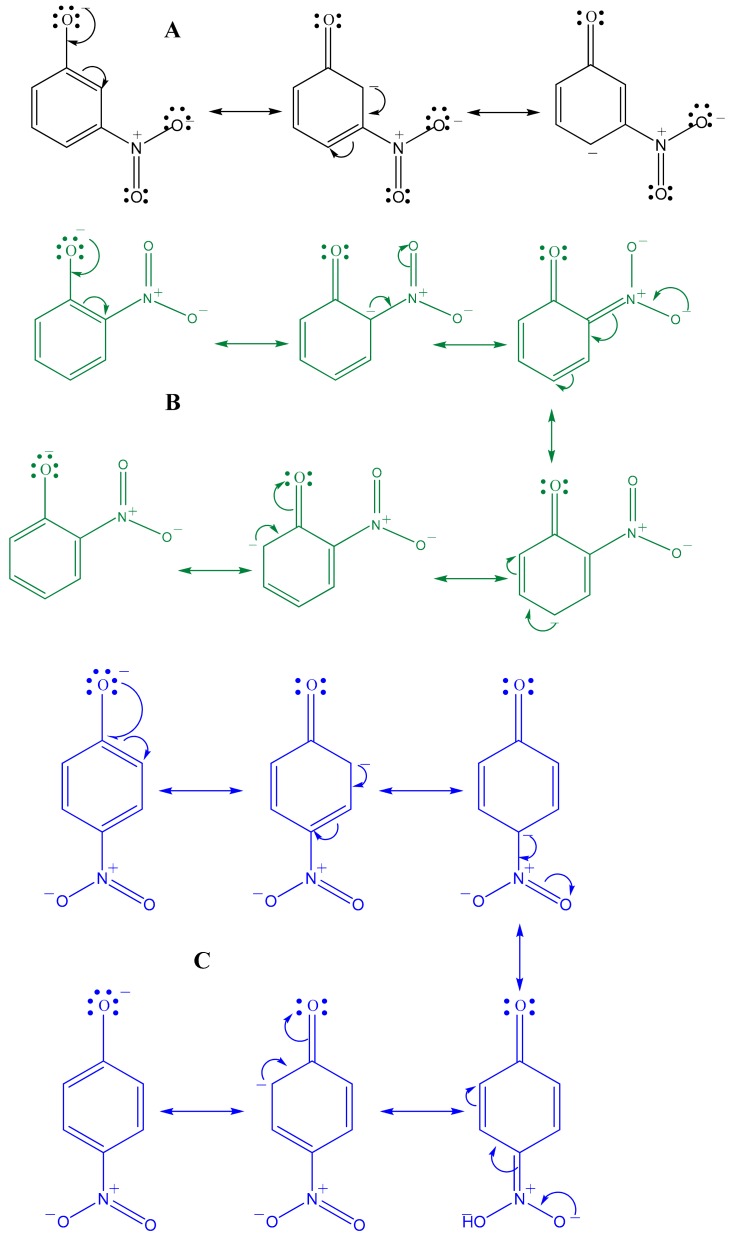
Electronic conjugation in the three nitrophenol isomers: (**A**) is 3-NP, (**B**) is 2-NP, and (**C**) is 4-NP.

However, catalytic reduction tests done on the para-nitrophenol (4-NP) and the ortho-nitrophenol (2-NP) show a very low efficiency of our catalyst, as the reduction reaction takes more than 48 h. The catalytic reduction was possible only for the 3-NP. This can be due to the difference between the three isomers where the 3-NP is being more basic (pKa = 8.3), due to the conjugation across the ring where the negative charge appears in ortho and para positions. For the 2-NP and 4-NP isomers, the negative charge appears only at the meta position (pKa = 7.3 and 7.2 respectively) (Figure 6).

**Figure 7:**
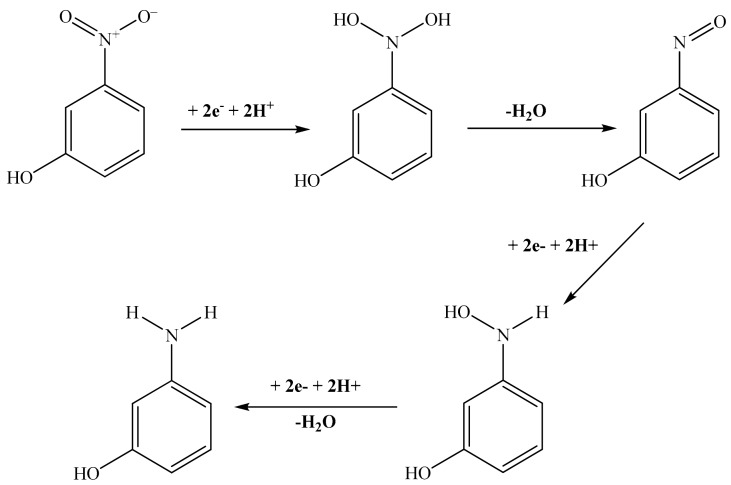
Proposed reduction mechanism of the 3-NP to 3-AP by NaBH_4_ in the presence of the prepared zinc molybdate.

On the other hand, in the case of the 3-NP, the electrons of the nitro group did not contribute in the conjugation, which makes them available, and makes the nitro group more reactive. A mechanism can be hypothetically suggested for the reduction of the 3-nitrophenol by NaBH_4_ in the presence of zinc molybdate catalyst. A mechanism with three reduction steps needing six electrons and dehydration steps is indicated in Figure 7.

This mechanism is based on the adsorption of the reducing agent that will be dissociated, liberating electrons that will reduce the adsorbed nitrophenol molecules. In fact, the zinc molybdate nanoparticles (ZnMoNPs) dissociated the BH_4_^−^ to form ZnMoNPs-H and ZnMoNPs-BH_3_^−^ as reactive intermediates (Equation (8)) [58]. Afterward, the 3-nitrophenol will be reduced as indicated in (Equations (9) and (10)).

2 ZnMoNPs + BH_4_^−^^↔^ ZnMoNPs-H + ZnMoNPs-BH_3_^−^ (8)

6 ZnMoNPs-H + 3-NP → 3-AP + 6ZnMoNPs + 2 H_2_O (9)

6 ZnMoNPs-BH_3_^−^ + 3-NP + 6 H^+^ →3-AP + 6 ZnMoNPs + 6 BH_3_ + 2 H_2_O (10)

### 3.4. Zinc Molybdate as Sorbent for Methylene Blue Removal

#### 3.4.1. Effect of Initial Dye Concentration

**Figure 8:**
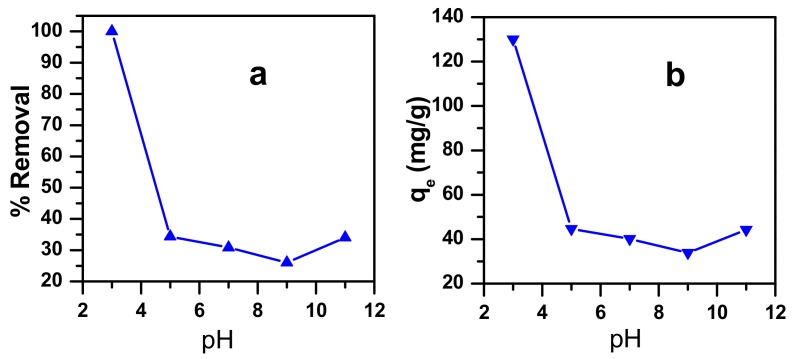
Effect of pH on MB dye adsorption onto zinc molybdate (**a**) removal efficiency (**b**) adsorption capacity at equilibrium.

**Figure 9:**
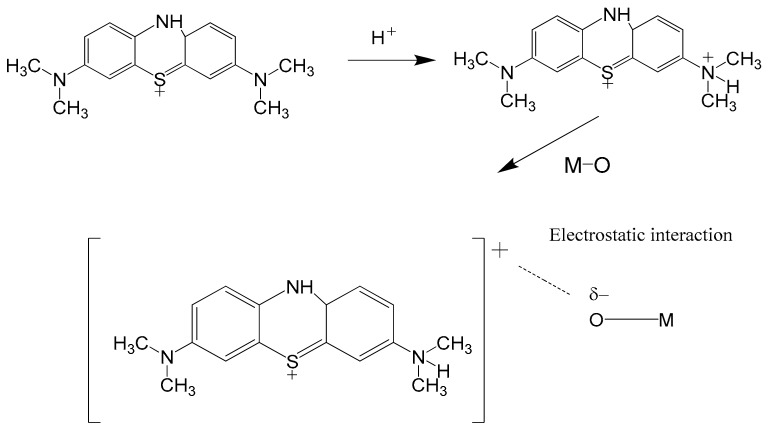
Schematic mechanism of the MB adsorption on the zinc molybdate nanoparticles surface.

The adsorption of cationic dye molecules is affected significantly by its pH, which is an important factor. The experiments were carried out in the pH range of 3–11, and the results show that the best condition for high removal of methylene blue (MB) is at pH = 3 (Figure 8). This result suggests an important role of the acidic media. Based on this, a mechanism can be proposed where, in the first step, protonation of the MB gives positively charged ammonium entities (–N^+^). In the second step, an electrostatic interaction occurs between these ammonium entities and the oxygen atoms of the zinc molybdate catalyst, that permits the adsorption of MB on the catalyst surface (Figure 9).

**Figure 10:**
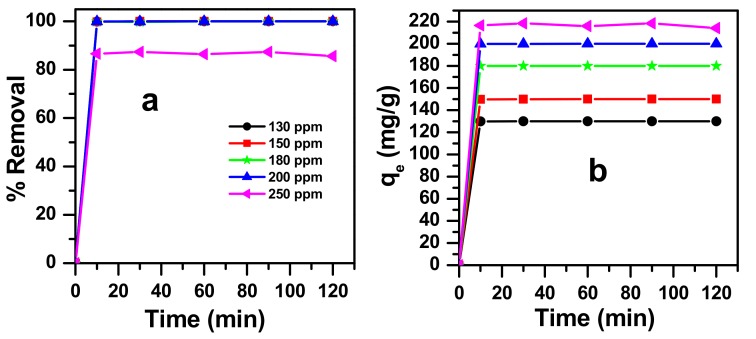
Effect of contact time on MB dye adsorption onto zinc molybdate at pH = 3 (**a**) removal efficiency (**b**) adsorption capacity at equilibrium.

The relationship between the dye concentration and the active sites available on the adsorbent surface affects the initial dye concentration. In fact, the effect of contact time adsorption of MB from an aqueous phase onto zinc molybdate surface was investigated at different contact time intervals in the range of 0 to 120 min. The results in Figure 10 show that the removal of MB by zinc molybdate reaches a maximum value of 100% at about 10 min, with a concentration range of 130 ppm to 200 ppm. However, at a concentration higher than 200 ppm, the percentage removal, after 120 min, reached 87% for 250 ppm. On the other hand, the results obtained for the adsorption capacity at equilibrium increases from 130 mg/g to 218 mg/g, with an increase in the initial dye concentration from 130 mg/L to 250 mg/L. Once the sites of the surface adsorbent were totally filled with MB dye molecules, there were no more surface adsorbent sites available for binding, and hence, the maximum adsorption was reached at this point [59].

#### 3.4.2. Adsorption Isotherm

The adsorption data are analyzed by proper adsorption isotherm models test. Freundlich and Langmuir isotherms are applied to the gained data of MB adsorption onto zinc molybdate. The Langmuir isotherm explains the adsorption of adsorbate on homogeneous adsorbent, as well as the monolayer adsorption, where there are no interactions between the adsorbate molecules. The Langmuir model linear equation is represented as follows [60]:

(11) $$\;\frac{C_{e}}{q_{e}}=\;\frac{1}{q_{m}K_{L}}+\frac{C_{e}}{q_{m}}$$

where *K_L_* (L·mg^−1^) is the Langmuir constant, which is related to the energy of the adsorption, and q_m_ (mg·g^−1^) is the maximum amount sorbed when the monolayer is complete.

The following equation defines the dimensionless constant, R_L_:

(12) $$\;R_{L}=\frac{1}{1+K_{L}C_{i}}$$

where C_i_ is the initial dye concentration (mg·L^−1^) and K_L_ is the Langmuir constant. The R_L_ value shows that the Langmuir isotherm is linear (R_L_ = 1), irreversible (R_L_ = 0), favorable (0< R_L_ < 1), and unfavorable (R_L_ > 1) [53]. Adsorption of adsorbate on a heterogeneous adsorbent is best described in the Freundlich isotherm model. The Freundlich linear model equation is shown as follows [61]:

(13) $$\;{\mathrm{Lnq}}_{e}={\mathrm{Lnq}}_{F}+\frac{1}{n}{\mathrm{LnC}}_{e}$$

where n and q_F_ are isotherm constants which indicate the intensity of the adsorption and adsorption capacity, respectively.

**Figure 11:**
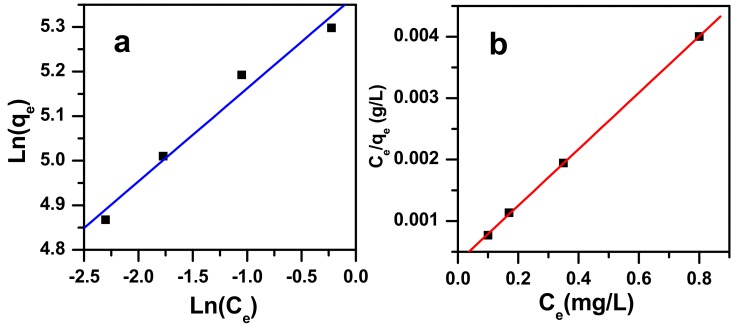
Freundlich (**a**) and Langmuir (**b**) isotherms for methylene blue dye adsorption onto zinc molybdate nanocatalyst at pH = 3 at room temperature.

The Freundlich and Langmuir adsorption isotherms of zinc molybdate nanoparticles for the removal of MB dye molecules are shown in Figure 11. The adsorption parameters of Freundlich and Langmuir for zinc molybdate are calculated and listed in Table 2.

In this study, comparing the correlation coefficient values (*R*^2^) for Langmuir and Freundlich isotherms, it has been found out that the Langmuir isotherm best fits the adsorption data rather than the Freundlich isotherm. Therefore, adsorption of the MB dye on zinc molybdate forms the adsorbate monolayer, which takes place on homogeneous adsorption sites. The surface area of the zinc molybdate adsorbent is 18.9 m^2^/g, and a pore size of 297 Å, and hence, this reaches a maximum adsorption capacity value.

#### 3.4.3. Desorption Isotherm

**Figure 12:**
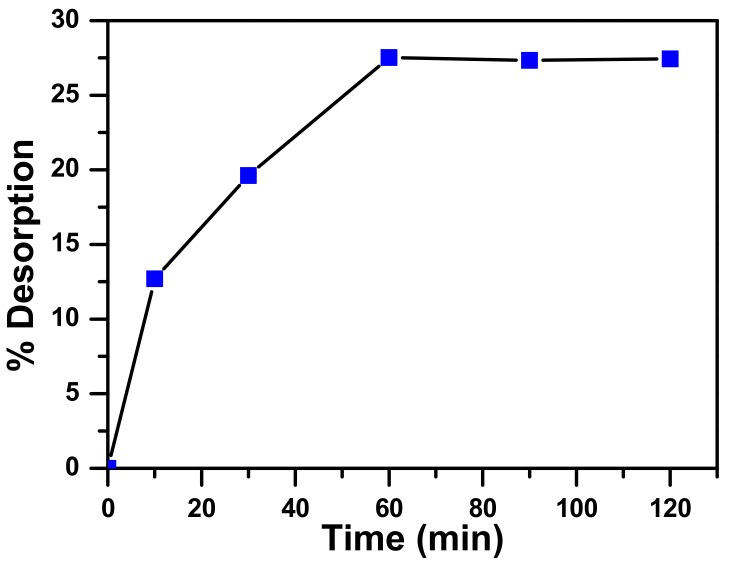
Rate of MB desorption from zinc molybdate after adsorption from MB 130 ppm solution at ambient temperature.

The rate of desorption of MB from the catalyst after its adsorption vs time is presented in Figure 12. The results shown that the equilibrium in these conditions was reached after one hour of contact time, leading to 27% of desorption. These results suggested that both of physisorption and chemisorptions take place in the same time. What was removed from the catalyst was the MB adsorbed by physisorption.

## 4. Conclusions

The synthesized zinc molybdate nanoparticles show high efficiency for two important applications in aqueous solutions, namely the removal of methylene blue and the reduction of 3-NP. Therefore, it can be concluded that the studied zinc molybdate is an essential catalyst as a nominee for the reduction of nitro functional groups in an amino group, and an efficient nanoadsorbent for the removal of methylene blue dye via the adsorption technique.

## Figures and Tables

**Table 1:** Reaction time comparison for the reduction of 3-NP by ZnMoO_4_ with previously reported nanocatalysts.

Catalyst	Type	Concentration of NP (mol/L)	Reaction Time (min)	References
ZnMoO_4_	Nanoparticles	2 × 10^−4^	1 for 3-NP	This work
Fe_2_(MoO_4_)_3_	Nanoparticles	2 × 10^−4^	6 for 3-NP	[39]
CuFe_2_O_4_	Nanoparticles	3.6 × 10^−5^	5 for 3-NP	[53]
NiFe_2_O_4_	Nanoparticles	3.6 × 10^−5^	36 for 3-NP	[53]
NiMoO_4_	Nanoparticles	2 × 10^−4^	3 for 3-NP	[54]
CuO/γAl_2_O_3_	Nanocomposites	2.9 × 10^−5^	20 for 3-NP	[55]
CoMoO_4_	Nanoparticles	2 × 10^−4^	1 for 3-NP	[56]
Ni/C black	Nanocomposites	5.0 × 10^−4^	15 for 3-NP	[57]

**Table 2:** Isotherm parameters for the adsorption of MB dye onto zinc molybdate surface at pH = 3 in room temperature.

Langmuir				Freundlich		
q_m_ (mg/g)	K_L_ (L/mg)	*R* ^2^	Range R_L_	q_F_ (mg^1-1/n^/L^1/n^/g)	1/n	*R* ^2^
217.86	13.80	1	0.0003–0.0006	215.10	0.21	0.97
